# Autophagy Benefits the Replication of Egg Drop Syndrome Virus in Duck Embryo Fibroblasts

**DOI:** 10.3389/fmicb.2018.01091

**Published:** 2018-05-29

**Authors:** Xueping Wang, Xuefeng Qi, Bo Yang, Shuying Chen, Jingyu Wang

**Affiliations:** College of Veterinary Medicine, Northwest A&F University, Yangling, China

**Keywords:** egg drop syndrome disease virus, autophagy, virus replication, autophagic flux, DEF cells

## Abstract

Egg drop syndrome virus (EDSV) is an economically important pathogen with a broad host range, and it causes disease that leads to markedly decreased egg production. Although EDSV is known to induce apoptosis in duck embryo fibroblasts (DEFs), the interaction between EDSV and its host needs to be further researched. Here, we provide the first evidence that EDSV infection triggers autophagy in DEFs through increases in autophagosome-like double-membrane vesicles, the conversion of LC3-I to LC3-II, and LC3 colocalization with viral hexon proteins. Conversely, P62/SQSTM1 degradation, LC3-II turnover, and colocalization of LAMP and LC3 confirmed that EDSV infection triggers complete autophagy. Furthermore, we demonstrated that inhibition of autophagy by chloroquine (CQ) and 3-methyladenine (3MA) or RNA interference targeting ATG-7 decreased the yield of EDSV progeny. In contrast, induction of autophagy by rapamycin increased the EDSV progeny yield. In addition, we preliminarily demonstrated that the class I phosphoinositide 3-kinase (PI3K)/Akt/mTOR pathway contributes to autophagic induction following EDSV infection. Altogether, these finding lead us to conclude that EDSV infection induces autophagy, which benefits its own replication in host cells. These findings provide novel insights into EDSV–host interactions.

## Introduction

Egg drop syndrome (EDS) is one of the most economically important diseases in the poultry industry, and it has wide host range, such as turkey breeder flocks, healthy laying birds, quail, and geese (Brugh et al., 1984; Bidin et al., 2007; Hafez, 2011). EDS is characterized by healthy laying hens apparently changing to produce eggs of poor quality, with clinical signs including soft, thin-shelled eggs, and shell-less eggs (Huang J. et al., 2015). EDS was first reported in Holland in 1976, and the infection then spread throughout the world and more recently appeared in China (Baxendale, 1978; McFerran and Smyth, 2000; Ezeibe et al., 2008; Fu et al., 2013). The causative agent, egg drop syndrome virus (EDSV) belong to the genus Atadenovirus within the family Adenoviridae, which are non-enveloped viruses with an approximately 30–35 kb linear double-stranded DNA genome (Fu et al., 2013; Larson et al., 2015). The adenovirus size range is 75–80 nm, and the replicative cycle is divided into two phases: early protein expression (E1–E4) and late protein expression (L1–L5) (Larson et al., 2015). Accumulating evidence has demonstrated that adenoviral proteins E1A and E1B play an essential role in establishing a productive virus infection by regulating cell proliferation, apoptosis, and autophagy (Kirn, 2000; Dobbelstein, 2004; Rodriguez-Rocha et al., 2011). In addition, hexon is the main building block of the adenovirus capsid. In recent years, numerous studies have revealed that hexon has a role in receptor binding, entry into the cell and apoptosis (Solanki et al., 2016; Yan et al., 2016; Qi et al., 2017). Despite the elucidation of some aspects of EDSV pathogenesis, the underlying mechanism of EDSV replication is still largely unknown.

Autophagy is an evolutionarily conserved process in eukaryotes that maintain cellular homeostasis, including metabolic balance (Klionsky, 2007; Kroemer et al., 2010). Autophagic activity is known to be controlled by a series of autophagy-related genes (Atg). Up to now, over 30 specific genes that take part in autophagy pathways have been identified in yeast (Klionsky et al., 2003; Milan et al., 2015). Upon autophagy induction, oncolytic adenoviruses activate a series of autophagy-related biomarker proteins, such as autophagy related microtubule-associated protein 1 light chain 3 (ATG8/LC3), Atg5 and Atg12, which form a complex that accumulates at the isolation membrane, and the polyubiquitin binding protein p62/SQSTM1 (p62), which assists in assessing autophagic flux because of the functional link between LC3 and the ubiquitinated substrates degraded by the autophagy pathway (Rodriguezrocha et al., 2011). Although autophagy was originally recognized as a protective function in the survival response of cells to a variety of extracellular and intracellular stimuli, it also plays a role in pathogen infection (Shintani and Klionsky, 2004; Levine and Kroemer, 2008; Deretic, 2010). Increasing evidence indicates that the autophagosomal structures induced by many RNA or DNA viruses may serve as platforms for viral replication (Suhy et al., 2000; Wong et al., 2008; Huang et al., 2009). In particular, recent research findings show that oncolytic adenovirus-induced autophagy promotes virus replication (Rodriguezrocha et al., 2011). However, the extent to which autophagy is induced during EDSV infection remains largely unknown.

The aim of the present study was to elucidate whether autophagy can be induced in permissive duck embryo fibroblasts (DEFs) during EDSV infection. By regulating autophagy via pharmacological treatment and RNA interference, we demonstrate that autophagy plays a positive and critical role in EDSV replication. In addition, we preliminarily revealed that the P13K/Akt/mTOR signaling pathway contributes to triggering autophagy in EDSV-infected host cells. A better comprehension of the interactions between EDSV and host autophagic responses will provide new insights into viral pathogenesis and antiviral drug development.

## Materials and Methods

### Cells and Viruses

Duck embryo fibroblasts were obtained from 11- to 12-day-old duck embryos as previously described (Huang W.R. et al., 2015), DEFs were maintained in high-glucose Dulbecco’s modified Eagle’s medium (DMEM, Gibco, United States) supplemented with 10% fetal bovine serum (Gibco, United States) at 37°C in 5% CO_2_. The EDSV127 strain (GenBank: Y09598) was obtained from the Green Square Biological Engineering Company (Shanxi, China) and was propagated in the allantoic fluid of 10-day-old specific-pathogen-free embryonated duck eggs at 38.5°C for 120 h. Virus titers for DEFs were determined on the basis of the 50% tissue culture infective dose (TCID_50_)/mL using the Reed–Muench method. The EDSV127 strain was at a titer of 10^6.1^/0.1 mL. To obtain a replication-incompetent EDSV strain, virus suspensions (2 mL) were irradiated with 254 nm UV light for 1 h. The lack of replication of UV-inactivation of EDSV was confirmed in DEFs. pcDNA3.1-hexon plasmids, or pCDNA3.1-control plasmids were previously established by our team (Qi et al., 2017) and kept in our laboratory. All experiments related to EDSV were done in a P2 biosafety laboratory and strictly carried out according to the Laboratory Biosafety Manual in our laboratory.

### Viral Infection and Cell Treatment

Duck embryo fibroblasts in 6-well plates were infected with EDSV at a multiplicity of infection (MOI) of 1 at 37°C (**Supplementary Figure S1**). Following a 2 h adsorption, unattached viruses were removed by aspiration, and the cells were washed three times with sterile PBS (pH 7.4) and incubated at 37°C in DMEM supplemented with 2% FBS until samples were harvested at different times. For autophagy induction, the DEFs were pretreated with rapamycin (Rapa: 100 nM; R0395, Sigma) dissolved in DMSO (D2650, Sigma) for 2 h, followed by a 2 h absorption of EDSV. The cells were cultured in fresh medium in the presence of 100 nM rapamycin until being harvested. For autophagy inhibition experiments, the DEFs were treated with chloroquine (CQ: 50 μM; C6628, Sigma) dissolved in PBS for 2 h prior to virus infection. In addition, cells were treated with 3-methyladenine (3MA: 5 mM; M9281, Sigma) or 740Y-P (25 μM; ApexBio Technology, Houston, TX, United States) dissolved in DMSO for 2 h prior to virus infection. Then, the DEFs were infected with EDSV at an MOI of 1. The inoculum was removed and cells were washed twice with PBS, and the cells were then incubated in fresh medium containing rapamycin (100 nM) or 3MA (5 mM) until harvesting of the cells or the culture medium. Moreover, the same amount of dimethyl sulfoxide (DMSO) was added to separate cells as the control group.

### Cell Viability Detection

For cell viability experiments, the optimal concentration of the corresponding drug used in this experiment was determined by the Cell Counting Kit-8 (96992, Sigma) assay according to the manufacturer’s instructions. In brief, 1 × 10^4^ DEFs were seeded in each well of 96-well culture plates, treated with each drug at different concentrations and then cultured for 24 h. Then, according to existing experimental procedures, the medium was removed and replaced with fresh medium containing CQ (25, 50, or 100 μM), rapamycin (50, 100, or 300 nM), 740Y-P (10, 25, or 50 μM), or 3MA (1, 5, or 10 mM) and incubated for 24 h. Premixed WST-8 cell proliferation reagent (10 μl) was then added to each well and incubated at 37°C for 4 h. Cell viability was evaluated by the measuring the absorbance at 450 nm, which was recorded using a 96-well plate reader (Bio-Rad Laboratories, Inc.).

### Transmission Electron Microscopy (TEM)

Duck embryo fibroblasts were mock infected or infected with EDSV at an MOI of 1 for 2 h. Then, the cells were cultured in 2% FBS for 36 h and were then washed three times with PBS (pH 7.4) and scraped free. DEFs were then collected by centrifugation at 1000 ×*g* for 10 min. The cell pellets were fixed with 2.5% glutaraldehyde/0.1 M phosphate buffer solution overnight at 4°C, washed three times in phosphate buffer solution for 10 min, and post-fixed with 1% osmium tetroxide at 4°C for 1 h. Following dehydration in a graded series of ethanol solutions, samples were embedded in a Spurr’s plastic resin. Finally, the autophagosome-like vesicles were examined under a Hitachi HT-7700 transmission electron microscopy (TEM) (Hitachi High Technologies, Co., Japan).

### Western Blot Analysis

Duck embryo fibroblasts infected with EDSV or treated with a drug were harvested at the indicated time, and cell samples were lysed with RIPA lysis buffer (P0013, Beyotime, Beijing, China). The lysates were briefly sonicated and cleared by centrifugation at 12,000 ×*g* for 5 min at 4°C. Protein samples were diluted in 5× SDS-PAGE loading buffer and were heated at 100°C for 5 min; after centrifugation at 12,000 ×*g* for 5 min, the samples were then separated on 12% SDS-PAGE gels and transferred to nitrocellulose membranes (Millipore, ISEQ00010). After incubation in blocking buffer (5% non-fat dry milk solution containing 0.05% Tween-20) at room temperature for 2 h, membranes were incubated overnight with the primary antibodies rabbit anti-LC3B (L7543, Sigma), rabbit anti-p62/SQSTM1 (P0067, Sigma), rabbit anti-ATG-7 (A2856, Sigma), and antibodies for PI3-k (#4257), phospho-PI3kinase p85 (Tyr458) (#4228), Akt (#4691), phospho-Akt (Ser473) (#9271), mTOR (#2983), phospho-mTOR (ser2448) (#2971) were purchased from cell signaling Technology, Inc. (Danvers, MA, United States). After thorough washing with Tris-buffered saline with Tween (TBST), the membranes were reacted with corresponding HRP-conjugated secondary antibodies (LI-COR Biosciences) for 2 h at room temperature. The target protein bands were visualized by an enhanced chemiluminescence detection kit (Thermo Fisher Scientific) with the ECL Plus western blot detection system (Amersham Biosciences, Piscataway, NJ, United States).

### Cell Transfection

Duck embryo fibroblasts were seeded in 12-well plated grown to 80% confluence were transfected with pCDNA3.1-hexon or an empty vector (pCDNA3.1-control). Four milligrams of expression plasmids combined with 10 μl Lipofectamine 2000 reagent (Thermo, Lithuania) was added to the cells according to the manufacturer’s instructions. Twenty-four hours post-transfection, the cells were seeded in 6-well plates for immunoblotting or cell viability assay.

### RNA Interference

Duck embryo fibroblasts were seeded in six-well cell culture plates. When cells had grown to 60 to 70% confluence, they were transiently transfected with ATG7 or scrambled siRNA with Lipofectamine 2000 (11668-27, Invitrogen) as previously described (Zhang et al., 2011). We used western blotting to test the interference efficiency of ATG7; scrambled siRNA was used as a negative control.

## Results

### EDSV Infection Upregulated the Levels of Autophagy in DEFs

**FIGURE 1:**
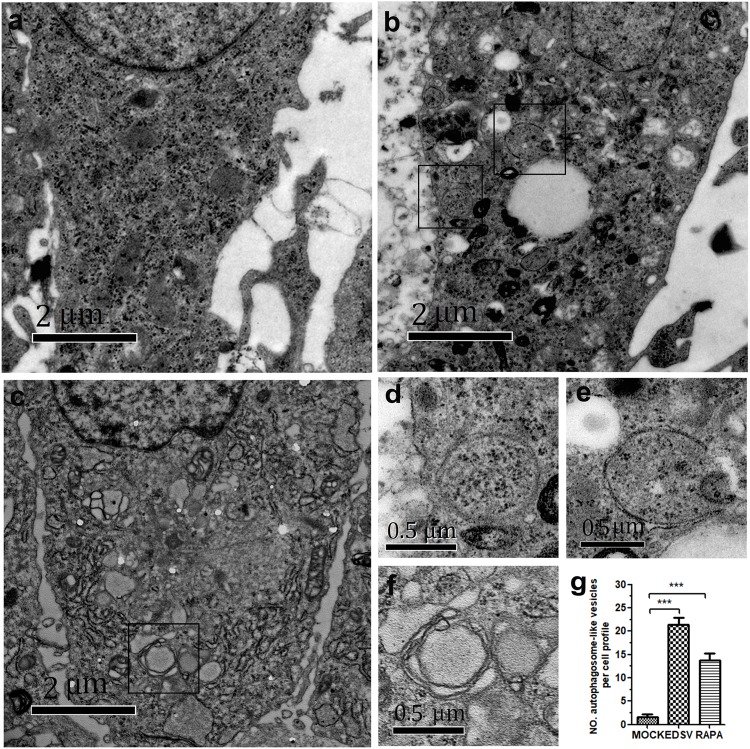
Egg drop syndrome virus (EDSV) infection triggers *autophagosome-like vesicles formation* in duck embryo fibroblasts (DEFs). **(a)** TEM observation. DEFs were mock-treated for 36 hpi, **(b)** infected with EDSV at an MOI of 1 for 36 hpi or **(c)** treated with rapamycin for 36 hpi and then processed for observation. Scale bar: 2 μm. **(d,e)** High magnification images of the typical autophagosome-like vesicles in EDSV-infected and **(f)** rapamycin-treated cells are shows. Scale bar: 0.5 μm. **(g)** The number of autophagosome-like vesicles per cell profile in mock- and EDSV-infected DEFs and rapamycin-treated DEFs were quantified. The results are shown as the mean ± SEM (*n* = 3). ^∗^*P* < 0.05; ^∗∗^*P* < 0.01; ^∗∗∗^*P* < 0.001.

To investigate whether autophagy is induced in DEFs by EDSV infection, the cells were mock infected or infected with the EDSV-127 strain or DEFs were treated with rapamycin for 36 hpi, and they were then observed through TEM. As shown in **Figure 1a**, double-membrane vesicles were rarely observed in mock-infected cells, whereas a large number of single-membrane vesicles and double-or single-membrane vesicles were observed in the cytoplasm of EDSV-infected DEFs (**Figure 1b**). These double-membrane structures were similar to the autophagosomes in DEFs induced by rapamycin, a well-known autophagy inducer, suggesting that the autophagosomes were induced in DEFs by EDSV infection (**Figure 1c**). Higher-magnification images of autophagosome-like vesicles in EDSV-infected cells are show in **Figures 1d,e,f**. Quantitative analyses showed that the number of autophagosome-like vesicles in the cytoplasm of EDSV-infected DEFs was significantly higher than the number when DEFs were treated with rapamycin and the number in mock-infected DEFs (**Figure 1g**).

**FIGURE 2:**
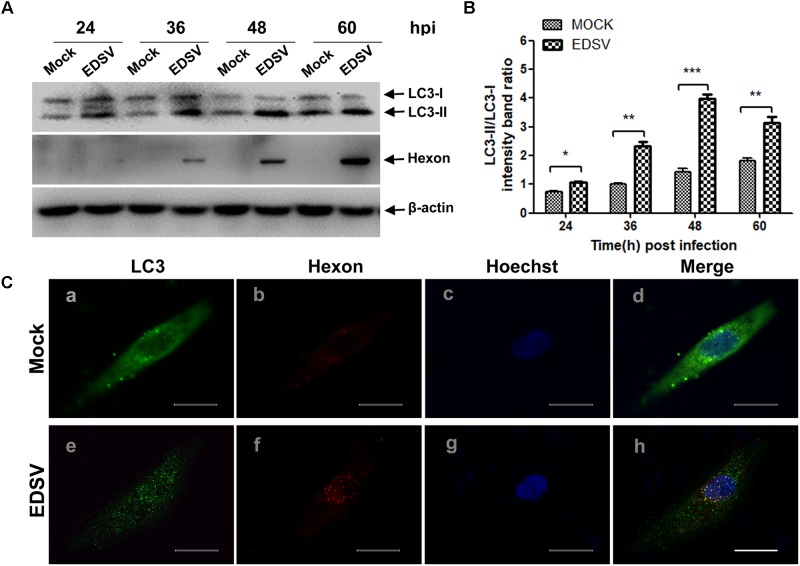
Modification of LC3 proteins during EDSV infection. **(A)** The significant conversion of LC3-l to LC3-ll was detected in mock-or EDSV-infected DEFs using an anti-LC3B antibody, and the expression of hexon from EDSV was also detected using anti-hexon antibodies. **(B)** The optical densities of each protein band were measured by densitometric scanning, and the optical density ratio of LC3-ll/LC3-l was calculated. The results are shown as the mean ± SEM (*n* = 3). ^∗^*P* < 0.05; ^∗∗^*P* < 0.01; ^∗∗∗^*P* < 0.001. **(C)** Confocal immunofluorescence microscopy images of mock-infected control and EDSV-infected DEFs. Mock- and EDSV-infected cells were fixed at 36 hpi and stained with antibodies against LC3 and EDSV hexon and then merged. Scale bar: 10 μm. Cell nuclei were counterstained with Hoechst. Fluorescence images were examined under a confocal laser scanning microscope. Scale bar = 10 μm.

Next, to further examine the autophagy induced by EDSV infection, we performed western blot assays to analyze the conversion of LC3-I to LC3-II. As shown in **Figure 2A**, we observed that LC3-II levels in EDSV-infected cells began to increase at 24 hpi, and LC3-l-to-LC3-II conversion was significantly greater in EDSV-infected DEFs than in mock-infected cells at the indicated time. The difference in the ratio of LC3-II/LC3-I between the EDSV-infected cells and the mock-infected cells was significant (*P* < 0.001) at 48 and 60 hpi (**Figure 2B**). Meanwhile, hexon proteins that specifically recognize EDSV were used to track the progression of infection. As shown in **Figure 2A**, increased viral levels were detected in EDSV-infected cells from 24 to 48 hpi.

In addition, EDSV infection led to a significant enhancement of punctate staining signals for LC3 distributed throughout the entire cytoplasm (**Figure 2C**), whereas the mock-infected DEFs exhibited no LC3 punctate accumulation. More importantly, signals of hexon protein from EDSV in EDSV-infected cells were colocalized with the punctate green fluorescent staining of LC3 (**Figure 2C**). Based on these experiment, we concluded that autophagy was significantly enhanced in DEFs upon EDSV infection.

### Increased Level of Autophagic Flux in EDSV-Infected DEFs

**FIGURE 3:**
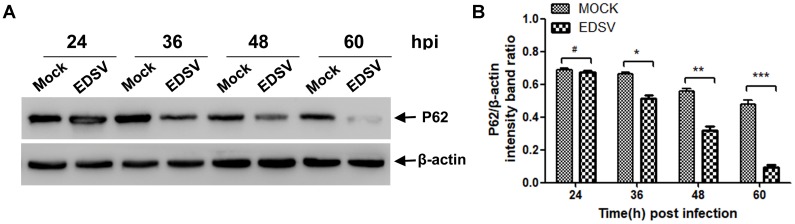
Egg drop syndrome virus infection enhances p62 degradation in DEFs. **(A)** A representative image of a western blot analysis of relative levels of p62 in mock- or EDSV-infected DEFs at indicated time points. **(B)** The optical densities of each protein band were measured by densitometric scanning, and the optical density ratio of p62/β-actin was calculated. The results are shown as the mean ± SEM (*n* = 3). ^∗^*P* < 0.05; ^∗∗^*P* < 0.01; ^∗∗∗^*P* < 0.001; ^#^Means no significant difference, *P* > 0.05.

P62 also known as sequestosome-1 (SQSTM1), is multifunctional protein that has been recognized as an indicator for assessing autophagic flux (Lippai and Low, 2014). As shown in **Figure 3A**, immunoblotting revealed significant progressive degradation of p62 in EDSV-infected cells compared with the unaltered p62 levels in mock-infected cells, suggesting that autophagosomes were able to fuse with lysosomes to degrade the cargos. The ratios of p62/β-actin shown in **Figure 3B** indicate that p62 reduction was notable at 48 hpi and peaked at 60 hpi.

**FIGURE 4:**
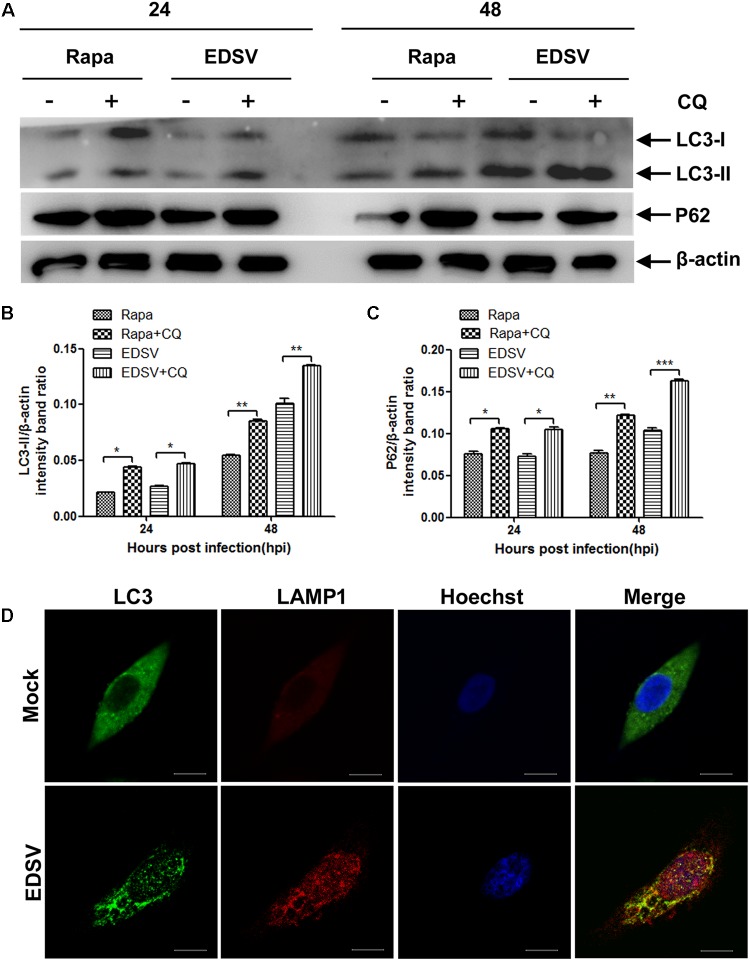
Egg drop syndrome virus infection enhances autophagic flux. **(A)** Western blot analyses of the effect of CQ treatment on the expression of both LC3-ll and p62 proteins in DEFs. Cell lysates were subjected to western blot analyses using antibodies against LC3, p62, and β-actin. Representative profiles are shown, and similar results were obtained in three independent experiments. **(B,C)** Analysis of changes in the relative amounts of both LC3-ll and p62 in rapamycin- pretreated and EDSV-infected DEFs in the absence and presence of CQ. **(D)** Representative images of colocalization of LC3 with the endosomal/lysosomal marker LAMP1 in mock- and EDSV-infected DEFs. Cell nuclei were counterstained with Hoechst. Fluorescence images were examined under a confocal laser scanning microscope. The results are shown as the mean ± SEM (*n* = 3). ^∗^*P* < 0.05; ^∗∗^*P* < 0.01; ^∗∗∗^*P* < 0.001. Scale bar = 10 μm.

Then, we used western blot-based measurements of LC3-ll turnover in the presence or absence of the lysosomal protease inhibitor chloroquine (CQ), which can inhibit the fusion of autophagosomes with lysosomes and leads to a marked accumulation of autophagic vacuoles, inducing intense LC3-II accumulation (Boya et al., 2005; Geng et al., 2010). As demonstrated in **Figures 4A,B** the accumulation of LC3-II and p62 was observed with CQ treatment, representing enhanced of autophagic flux at 24 and 48 hpi post-infection in EDSV-infected DEFs compared with the mock-treated cells.

Furthermore, we measured LC3 protein colocalization with lysosome-associated membrane protein 1 (LAMP1). As demonstrated in **Figure 4C**, the colocalization of LC3 and LAMP1 was detected in EDSV-infected DEFs, while mock-infected cells showed no overlap (**Figure 4D**). These results indicated that DEFs go through a complete autophagic process following EDSV infection.

### Virus Replication Is Required for EDSV Induction of Autophagy

**FIGURE 5:**
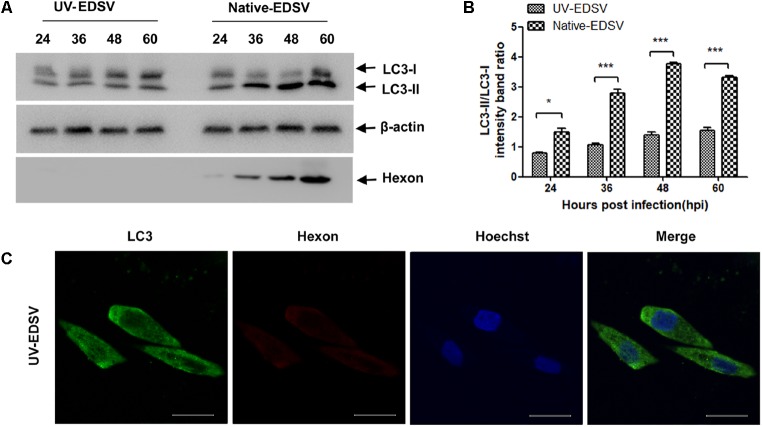
Western blot analysis of the ability of UV-inactivated EDSV to induce the conversion of LC3-l to LC3-ll in DEFs. **(A)** Cells infected with replication -competent EDSV or with UV-inactivated EDSV (UV-EDSV) at an MOI of 1. **(B)** Densitometric analysis of relative levels of LC3-II and LC3-I in EDSV- or UV-EDSV -infected DEFs. The results are shown as the mean ± SEM (*n* = 3). ^∗^*P* < 0.05; ^∗∗^*P* < 0.01; ^∗∗∗^*P* < 0.001. **(C)** Confocal microscopy analysis of the ability of UV-EDSV to induce autophagy in DEFs. Scale bar = 15 μm.

To reveal whether EDSV replication is necessary for the induction of autophagy, ultraviolet (UV)-irradiated EDSV was used to infect DEFs. As shown in **Figures 5A,B**, LC3 proteins in UV-inactivated EDSV-infected DEFs did not undergo conversion from LC3-l to LC3-ll, and the expression of hexon proteins was not detected in this group. In contrast, the levels of both LC3-I and LC3-II in DEFs indicated apparent conversion in DEFs inoculated with live EDSV, which was accompanied with an increase in hexon protein levels. Furthermore, the results of confocal microscopy further verified that UV-inactivated EDSV was not able to induce the LC3 colocalized with hexon proteins in EDSV-infected DEFs (**Figure 5C**). In brief, the results indicated that virus replication was necessary for induction of autophagy by EDSV infection.

### The Replication of EDSV in DEFs Is Enhanced by the Induction of Autophagy

To investigate whether the replication of EDSV in DEFs is enhanced by autophagy induction, DEFs, prior to virus inoculation, were treated with rapamycin, which can specifically induce the autophagy pathway by directly inhibiting mTOR (Cina et al., 2012). Cells were harvested at 24 and 48 hpi and then subjected to western blot analysis. Meanwhile, virus progeny were collected at the corresponding time points for titration.

**FIGURE 6:**
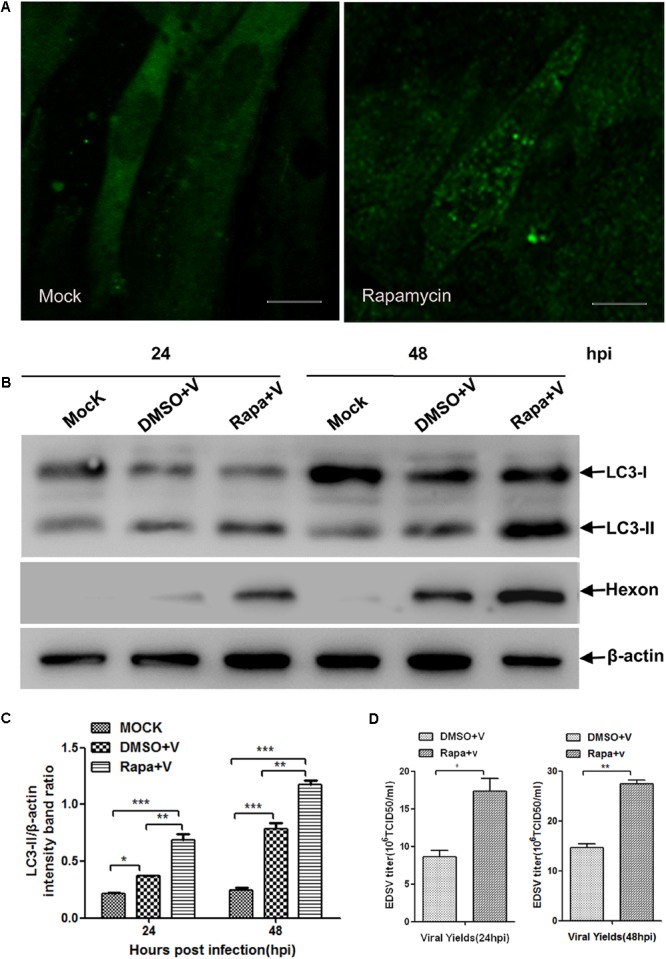
Autophagy induction enhanced the replication of EDSV. **(A)** A representative image from confocal immunofluorescence microscopy analysis of LC3 expression in EDSV-infected DEFs or DEFs treated with 100 nM rapamycin prior to EDSV infection. Scale bar = 10 μm. **(B)** Western blot analyses the cells infected with DMSO (mock), cells treated with rapamycin for 4 h and then infected with EDSV at an MOI of 1 (Rapa + v), cells treated with DMSO for 4 h and then infected with EDSV at an MOI of 1 (DMSO + v), which were harvested at 24 and 48 hpi. **(C)** Densitometric analysis of both LC3-ll and β-actin proteins in mock, Rapa + v, and DMSO + v groups. **(D)** Quantification of the yields of virus progeny in EDSV-infected DEFs pretreated with DMSO (DMSO+V) and in EDSV-infected cells pretreated with rapamycin (Rapa+v) were determined according to the TCID50/mL. The results are shown as the mean ± SEM (*n* = 3). ^∗^*P* < 0.05; ^∗∗^*P* < 0.01; ^∗∗∗^*P* < 0.001.

Compared with the mock-infected DEFs (**Figure 6A**), LC3 punctate staining increased in the DEFs treated with rapamycin (**Figure 6A**). Moreover, we further observed that LC3-ll exhibited an increasing trend in rapamycin-treated cells compared to the cells treated with DMSO (**Figures 6B,C**). These results show that rapamycin treatment enhanced autophagy in DEFs. Along with the enhancement of autophagy in EDSV-infected DEFs, the expression of EDSV hexon proteins was increased following treatment with rapamycin (**Figures 6B,C**). Additionally, we observed that the titers of EDSV in the infected DEFs treated with rapamycin were higher than those in the infected DEFs treated with DMSO (**Figure 6D**). These results suggest that autophagy enhanced the replication of EDSV in DEFs.

### The Replication of EDSV Is Reduced by the Inhibition of Autophagy

**FIGURE 7:**
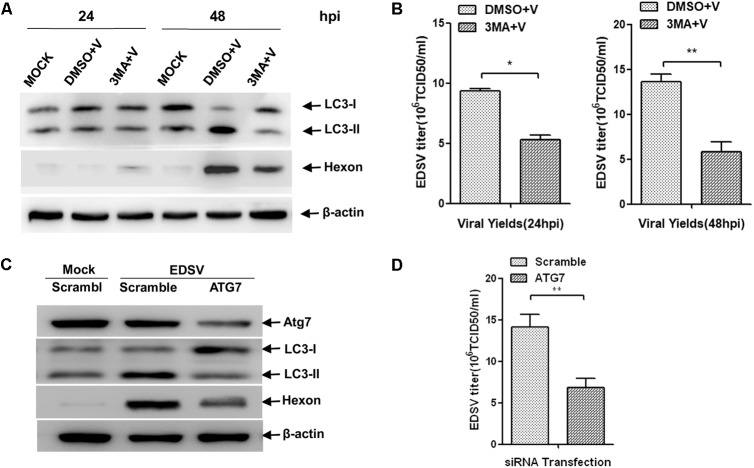
Inhibition of autophagy reduces EDSV replication in EDSV cells. **(A)** Western blot analysis of the effect of 3MA (5 mM) on the expression of both LC3 and hexon proteins in DEFs. Cells infected with DMSO (mock), cells infected with EDSV at an MOI of 1 (DMSO+V) and cells treated with 3MA for 4 h and then infected with EDSV an MOI of 1 (3MA+V) were harvested at 24 and 48 hpi. Cell samples were processed for western blot analysis, using antibodies against LC3, hexon, and β-actin, at 24 and 48 hpi. **(C)** Western blot analysis of the effect of ATG7 silencing on the expression of both LC3 and hexon proteins in DEFs. DEFs were transfected with either ATG7 siRNA (ATG7) or non-targeting siRNA (Scramble). Cell samples were processed for western blot analysis using antibodies against ATG7, LC3, hexon, and β-actin at 48 hpi. **(B,D)** Quantification of the yields of progeny virus in EDSV-infected DEFs were determined according to the TCID50/mL. The results are shown as the mean ± SEM (*n* = 3). ^∗^*P* < 0.05; ^∗∗^*P* < 0.01; ^∗∗∗^*P* < 0.001.

Since the replication of EDSV in DEFs is enhanced by the induction of autophagy, we may ask what effect autophagy inhibition will have on virus replication. 3MA is widely used to inhibit autophagy at the early stage by targeting class III phosphatidylinositol-3-kinase (P13k) (Seglen and Gordon, 1982). 3MA treatment not only reduced LC3-l/LC3-ll conversion but also reduction expression of hexon proteins and the yields of virus titers at 24 and 48 hpi (**Figures 7A,B**).

To further confirm the relationship between autophagy and EDSV replication. We depleted the essential endogenous ATG7, which encodes an E1-like enzyme that is essential to the formation of autophagic vacuoles (Kim et al., 1999), and knockdown of the ATG7 gene can seriously impair the autophagic pathway (Komatsu et al., 2005). As expected, transfection with ATG7-targeting siRNA resulted in an obvious decrease of endogenous Atg7 proteins at 48 hpi. Meanwhile, the expression of LC3-ll was slightly decreased (**Figure 7C**). In addition, we found that the expression of EDSV hexon proteins (**Figure 7C**) as well as the yield of EDSV progeny were significantly reduced (**Figure 7D**). These data support our hypothesis that autophagy induction could facilitate EDSV replication.

### EDSV Triggered Autophagy in DEFs via PI3K/Akt/mTOR Signaling Pathway

**FIGURE 8:**
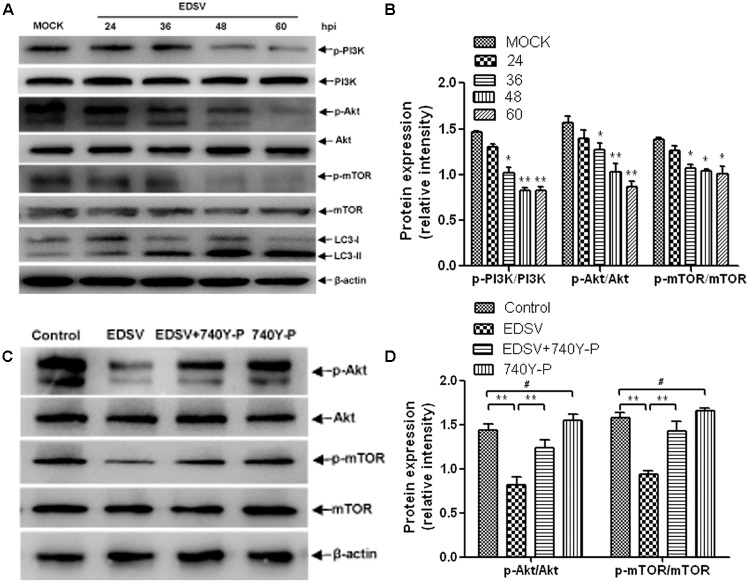
Egg drop syndrome virus induced autophagy in DEFs via the P13k/Akt/mTOR pathway. **(A)** DEFs infected with EDSV was harvested at the indicated time points post-infection and analyzed for the expression of p-P13k, P13k, p-mTOR, mTOR p-Akt, and Akt by western blot assay. **(C)** The levels of Akt, p-Akt, p-mTOR, and mTOR in DEFs treated with 0.1% DMSO (control group), EDSV, EDSV + 740Y-P (25 μM), 740Y-P (25 μM) were measured by western blot assay. **(B,D)** The optical densities of each protein band were measured by densitometric scanning, and the optical density ratios of p-P13k/P13k, p-mTOR/mTOR, and p-Akt/Akt were calculated. The results are shown as the mean ± SEM (*n* = 3). ^∗^*P* < 0.05; ^∗∗^*P* < 0.01; ^∗∗∗^*P* < 0.001, “#” means no significant difference, *P* > 0.05.

The role of the pI3K/Akt/mTOR pathway in induction of autophagy was examined in EDSV-infected DEFs. As shown in **Figure 8**. EDSV infection resulted in significant reductions in phosphorylation of p-pI3k, p-Akt, and p-mTOR in DEFs at indicated time points post-infection (**Figures 8A,B**). However, the expression of LC3-II was increased after EDSV infection (**Figures 8A,B**). To further verify the above findings, EDSV-infected DEFs were treated with or without 740Y-P, which is a PI3K activator that can lead to the upregulation of p-mTOR and p-Akt (Zhang et al., 2015). The results showed that the downregulation of p-mTOR and p-Akt was significantly recovered in the presence of 740Y-P (**Figure 8C**). These results indicate that EDSV reduces p-P13k, p-mTOR, and p-Akt protein phosphorylation in infected DEFs.

### Hexon Triggered Autophagy in DEFs via PI3K/Akt/mTOR Signaling Pathway

**FIGURE 9:**
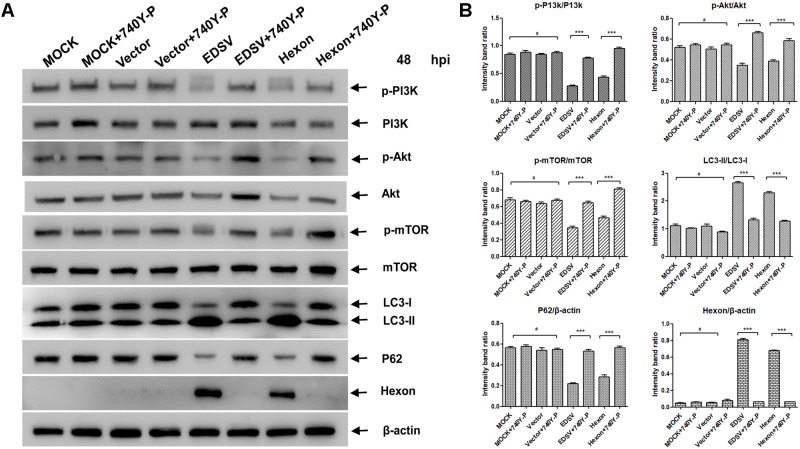
Hexon triggered autophagy in DEFs via the P13k/Akt/mTOR pathway. **(A)** Western blot analysis of the effect of 740Y-P (25 mM) on the expression of p-PI3k, PI3k, p-Akt, Akt, p-mTOR, mTOR, LC3, P62, and hexon proteins in DEFs. Cell lysates were subjected to western blot analyses using antibodies against p-PI3k, PI3k, LC3, p62, hexon, and β-actin. Representative profiles are shown, and similar results were obtained in three independent experiments. **(B)** The optical densities of each protein band were measured by densitometric scanning, and the optical density ratios of LC3II/I, P62/β-actin, hexon/β-actin, p-P13k/P13k, p-mTOR/mTOR, and p-Akt/Akt were calculated. The results are shown as the mean ± SEM (*n* = 3). ^∗^*P* < 0.05; ^∗∗^*P* < 0.01; ^∗∗∗^*P* < 0.001, “#” means no significant difference, *P* > 0.05.

In order to study whether hexon expression also induces autophagy in DEFs as similar as EDSV infection, we transfected DEFs with PCDNA-hexon. As shown in **Figure 9A**, the hexon protein expression levels increased in parallel with an increase in LC3-l-to-LC3-ll conversion, and P62 gradually decreased in hexon-transfected DEFs. These results show that EDSV hexon expression can induce autophagy in DEFs. To examined whether the pI3K/Akt/mTOR signaling pathway was associated with hexon-induced autophagy in hexon-transfected DEFs. As show in **Figures 9A,B**. The p-P13k, p-Akt, and p-mTOR phosphorylation protein expression levels was decreased in hexon-transfected DEFs at 48 hpi. To further verify the above findings, hexon-transfected DEFs were treated with 740Y-P or without 740Y-P. The results showed that the downregulation of p-P13k, p-mTOR and p-Akt was significantly recovered in the presence of 740Y-P (**Figures 9A,B**). Altogether, these results indicated that hexon not only induced autophagy, but reduced the phosphorylation of p-P13k, p-mTOR, and p-Akt in DEFs at 48 hpi.

### Modulation of Autophagy Activity With Autophagy Regulators Does Not Affect Cell Viability

**FIGURE 10:**
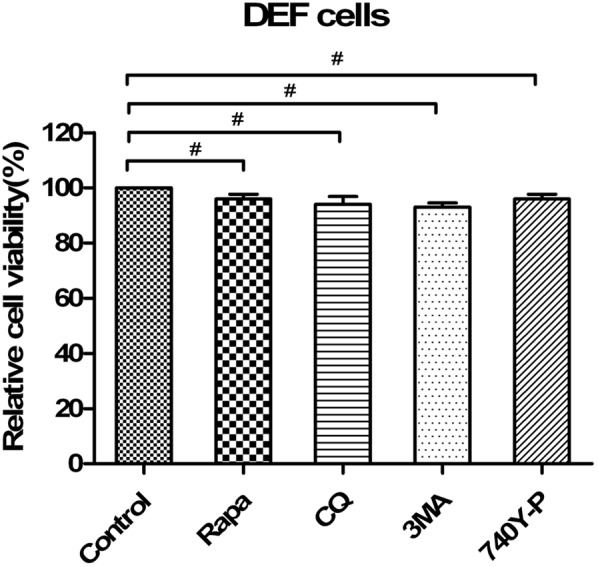
Pharmacological alterations had no effects on cell viability. DEF viability was determined by WST-8 cell proliferation assay after treatments with rapamycin (Rapa), chloroquine (CQ), 3-methyladenine (3MA) or 740Y-P or transfection with siATG7 for 48 h. Data are presented as the mean ± SEM of three independent experiments. “#” means no significant difference, *P* > 0.05.

Moreover, we tested whether high concentrations of drugs used to pharmacologically alter autophagy, including CQ treatment, 3MA treatment, rapamycin treatment and 740Y-P treatment, affected the capability of EDSV to replicate. Statistical analyses revealed no significant effects by Cell Counting Kit-8 assay (**Figure 10**).

## Discussion

As an economically important disease of the poultry industry, EDSV infection reduces egg production in chickens and induces an acute respiratory disease in goslings, leading to substantial economic losses. It is believed that ducks are a natural host of EDSV since EDSV and EDSV antibodies have been found repeatedly in various domesticated ducks, and global serologic evidence of egg drop syndrome (EDS) has been reported in a variety of wild waterfowl species (Cha et al., 2013a,b; Huang J. et al., 2015). DEFs are the natural target cells of EDSV, and they provide a valuable *in vitro* model reference to study host–virus interaction (Wang et al., 2015; Qi et al., 2017; Yin et al., 2017). Many researchers have mainly focused on adenovirus infections in human cells, and little is known about adenovirus infections in avian-derived cells. Huang J. et al., (2015) found that DEFs are suitable hosts for EDSV replication. Qi et al. (2017) study demonstrated that *in vitro* infection of DEFs with EDSV caused apoptosis, which may have been associated with virus replication. Recently, many researchers have documented that apoptosis has a complex interaction with autophagy or can even be induced simultaneously by pathogen infection (Gougeon and Piacentini, 2009; Xin et al., 2014). Thus, it is essential to resolve the question of whether EDSV infection in host cells triggers autophagy and what its role is in this process.

In this study, we demonstrated that EDSV triggers autophagy in DEFs, as shown by TEM, including the presence double-and single-membrane vesicles, indicating that autophagosomes were formed. Meanwhile, we monitored the conversion of LC3-l/LC3-ll in EDSV-infected DEFs by western blot analysis. In addition, we found that hexon proteins of EDSV were colocalized with LC3 proteins in the EDSV-infected DEFs, as shown by confocal microscopy analysis. Our findings provide evidence that EDSV infection is able to induce autophagy in DEFs. We also assessed whether EDSV infection of DEFs triggers complete autophagy. Complete autophagy involves the dynamic process of autophagosome synthesis, including autophagosome formation and maturation, and degradation of autophagosomes within lysosomes; thus, autophagic flux is a more accurate indicator of autophagy activity than autophagosome formation (Mizushima et al., 2010). Notably, the autophagic response to different viruses is not always the same. The autophagic flux induced by adenoviruses and duck enteritis virus is a complete autophagic response (Jiang et al., 2011; Yin et al., 2017). However, influenza A virus and Rotavirus, by blocking autophagosome degradation, induce an incomplete autophagic response (Gannage et al., 2009; Crawford et al., 2012). This implies that autophagic flux is virus-specific. To clarify whether infection with EDSV induces complete autophagy, we used several means to measuring autophagic flux, including monitoring p62 degradation after EDSV infection. The lysosome inhibitor CQ increased the levels of LC3-II and p62 in virus-infected cells. Furthermore, fluorescent LAMP1 puncta colocalized with LC3-positive puncta in EDSV-infected cells. These findings demonstrated that complete autophagic flux was triggered upon EDSV infection.

Egg drop syndrome virus infection can trigger complete autophagy in DEFs; however the role of the EDSV replication process in regulating autophagy is unclear. Autophagy is a balancing mechanism that maintains the homeostasis of cells. Autophagy commonly serves as a defense mechanism to clear viruses and reduce viral growth (Yakoub and Shukla, 2015). Nevertheless, growing evidence suggests that some DNA viruses such as hepatitis B virus, duck enteritis virus, and adenoviruses have evolved strategies to use autophagic vesicles for replication (Li et al., 2011; Rodriguezrocha et al., 2011; Yin et al., 2017). In further determining what role autophagy plays in the replication of EDSV, we observed that EDSV infection resulted in LC3-l/LC3-ll conversion, which continued to increase with infection time. These data demonstrate that the autophagy induced by EDSV infection may be closely correlated with viral replication. Notably, UV-inactivated EDSV does not trigger LC3-l/LC3-ll conversion in DEFs. This result was further confirmed by confocal microscopy, which showed that UV-inactivated EDSV did not result in LC3 colocalizing with hexon proteins in EDSV-infected DEFs. Therefore, it may be implied that EDSV replication is necessary during autophagosome formation. Then, we analyzed the specific effect of autophagy on EDSV infection by administering pharmacological regulators. Our data showed that rapamycin treatment not only upregulated the expression of viral hexon proteins but also increased the yield of EDSV progeny. We also demonstrated that inhibition of autophagy with 3MA or shRNA-based depletion of the essential autophagy protein ATG-7 downregulated hexon expression and decreased the yield of EDSV progeny. Meanwhile, the data from the CCK-8 assay indicated that pharmacological alterations of autophagy had no significant effects on cell viability following treatment with rapamycin, CQ or 3MA. Taken together, these findings reveal that induction of autophagy during EDSV infection promotes virus replication.

Although EDSV induces autophagy to help its replication, the exact mechanism underlying this process is unclear. Previous studies demonstrated that adenovirus proteins E1A and E1B play an essential role in the induction of pro-autophagic signaling pathways (Polager et al., 2008; Piya et al., 2011). Moreover, it has been shown that suppression of the mTOR signaling pathway by rapalogs such as rapamycin and everolimus enhances autophagic cell death in oncolytic adenoviral therapy (Yokoyama et al., 2008; Rajecki et al., 2009). It is well-known that the serine/threonine kinase mTOR in mammalian cells is a central regulator of autophagy. mTOR mainly functions downstream from the PI3K-Akt signaling pathway and plays important roles in negatively regulating autophagy (Jung et al., 2010; Kim et al., 2011; Cao et al., 2017). However, adenoviral and adenoviral-related protein regulation of autophagy through the PI3K/Akt/mTOR signaling pathway has rarely been reported. In our study, we observed that at the middle to late stages of EDSV infection, there was a reduction in p-P13K, p-Akt, and p-mTOR protein phosphorylation in infected DEFs, indicating a critical role of the PI3K/Akt/mTOR pathway in EDSV-triggered autophagy.

Increasing evidence indicates that PI3K activation is affected by many factors, such as different sources of virus strains, cell lines and different viral proteins, that may cause significant differences in the regulation of P13K signaling pathways (Shin et al., 2007; Ma et al., 2011; Huang W.R. et al., 2015; Xie et al., 2016). Some studies reported that adenovirus penton proteins interact with α_v_ integrins to promote adenovirus internalization and that this specifically involves mediation of PI3K activation (Li et al., 1998). However, previous studies of EDSV penton proteins are limited to serological surveys and the complete viral genome analysis and what role dose the penton proteins play during EDSV infection and replication are unknown (Rohn et al., 1997; Xiao et al., 2009). Therefore, some questions naturally arise, such as whether the penton protein promotes the internalization of EDSV and whether the process requires the downregulation or activation of PI3K. Moreover, the expression of E4 induced by E1A via cellular transcription factor E4F, participates in the activation of the PI3K/mTOR pathway and play an important role in adenovirus-infection (Raychaudhuri et al., 1987; O’Shea et al., 2005). Whereas, according to published researches, E1A cannot be found in EDSV (Qi et al., 2017). In terms of this, further study is needed to clarify how the lack of E1A proteins affects the function of other proteins or which proteins can replace E1A proteins and perform its function in EDSV-infected host cells. Hexon is the major protein of the virus capsid and is important for the uptake of adenovirus into cells. Given the findings that there is a temporal correlation between changes in autophagy and hexon expression levels observed in EDSV-infected DEFs, we asked whether hexon can induce autophagy during EDSV infection. In this study, we found that hexon protein expression levels gradually increased in parallel with LC3-l-to-LC3-ll conversion rates, and P62 levels gradually decreased in hexon transfected DEFs, as shown by western blot analysis. These results clearly support the hypothesis that EDSV hexon expression can induce autophagy in DEFs. In addition, we found preliminary verification that EDSV hexon transfection reduces p-P13k, p-mTOR, and p-Akt protein phosphorylation in infected DEFs.

To summarize, our findings suggest that autophagy is triggered in host cells upon EDSV infection or hexon transfection and that both are involved in the replication of EDSV, which implies that EDSV can utilize the autophagic pathway to sustain replication in host cells. In addition, from our current study, it was found that both EDSV and EDSV hexon protein can reduce the phosphorylation of p-P13K, p-Akt, and p-mTOR protein in infected DEF cells. Clearly, our understanding of the molecular mechanisms driving the interplay between EDSV and autophagy is still insufficient in several aspects; for example, how hexon proteins regulate the mechanism of EDSV-induced autophagy via PI3K/Akt/mTOR pathway remains unclear. Besides, it not yet clear whether other proteins or virulence factors in EDSV affect the process that hexon proteins regulate the autophagy through different mechanisms in infected DEFs. However, this basic data presented here are helpful for further exploring the pathogenesis related to the interaction between EDSV and host cells. This knowledge will provide novel insights into autophagy abrogation and may be used to help develop potential antiviral strategies or drugs against EDSV infection.

## Author Contributions

XW, XQ, and JW conceived and designed the experiments. XW performed the experiments, analyzed the data, and wrote the paper. JW contributed reagents, materials and analysis tools. XW, XQ, SC, and BY prepared the figures and tables.

## Conflict of Interest Statement

The authors declare that the research was conducted in the absence of any commercial or financial relationships that could be construed as a potential conflict of interest.

## Supplementary Material

FIGURE S1 **(A)** The DEFs cells were infected by EDSV at different MOI for 48 h. The cells were lyzed and western bolt were performed. **(B)** The optical densities of each protein band were measured by densitometric scanning, and the optical density ratios of LC3II/I were calculated. Data are presented as the mean ± SEM of three independent experiments. ^∗^*P* < 0.05; ^∗∗^*P* < 0.01; ^∗∗∗^*P* < 0.001. “#” means no significant difference, *P* > 0.05.
