# Supplementary figures and images for: Autophagy Benefits the Replication of Egg Drop Syndrome Virus in Duck Embryo Fibroblasts
Source: Front Microbiol. 2018 May 29;9:1091. doi: 10.3389/fmicb.2018.01091 (PMC5986908; doi:10.3389/fmicb.2018.01091)

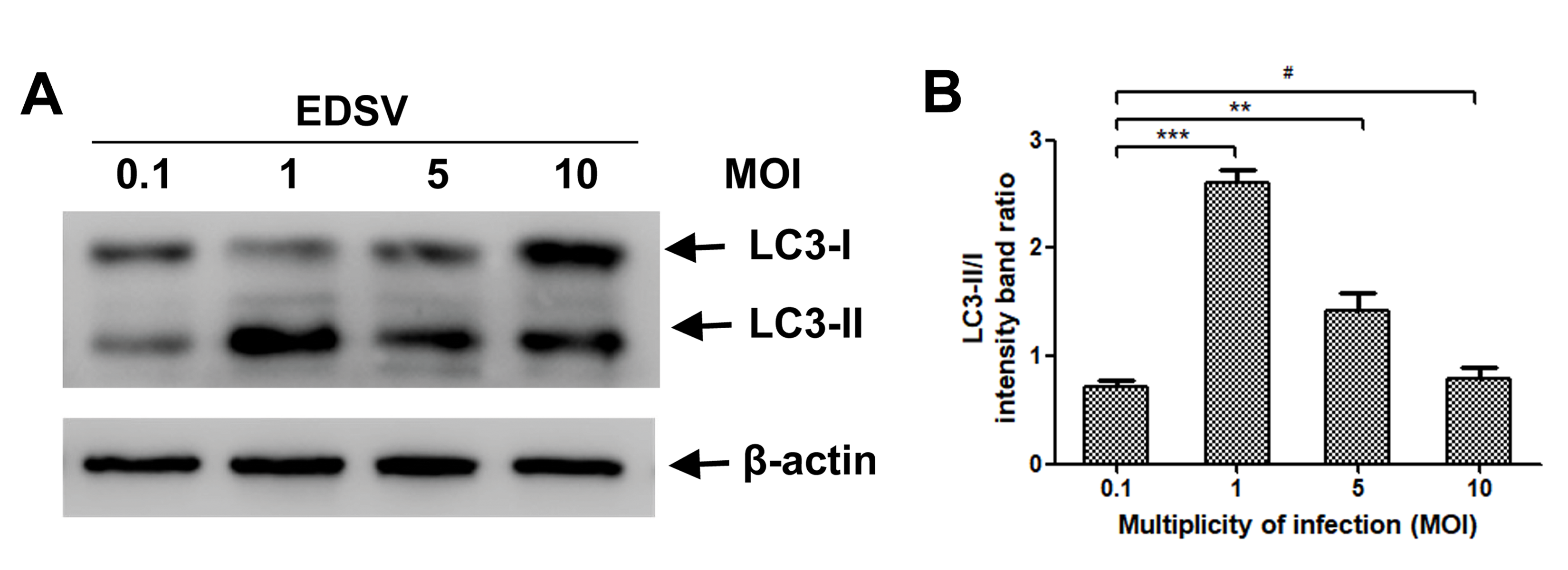

Supplement: FIGURE S1 — (A) The DEFs cells were infected by EDSV at different MOI for 48 h. The cells were lyzed and western bolt were performed. (B) The optical densities of each protein band were measured by densitometric scanning, and the optical density ratios of LC3II/I were calculated. Data are presented as the mean ± SEM of three independent experiments. ∗P < 0.05; ∗∗P < 0.01; ∗∗∗P < 0.001. “#” means no significant difference, P > 0.05. [file Image_1.TIF]
